# The Utility of Infliximab Therapeutic Drug Monitoring among Patients with Inflammatory Bowel Disease and Concerns for Loss of Response: A Retrospective Analysis of a Real-World Experience

**DOI:** 10.1155/2016/5203898

**Published:** 2016-11-10

**Authors:** Robert A. Mitchell, Constantin Shuster, Neal Shahidi, Cherry Galorport, Mari L. DeMarco, Gregory Rosenfeld, Robert A. Enns, Brian Bressler

**Affiliations:** ^1^St. Paul's Hospital, Department of Medicine, Division of Gastroenterology, University of British Columbia, Vancouver, BC, Canada; ^2^Department of Pathology and Laboratory Medicine, University of British Columbia, Vancouver, BC, Canada

## Abstract

*Background*. Infliximab (IFX) therapeutic drug monitoring (TDM) allows for objective decision making in patients with inflammatory bowel disease (IBD) and loss of response. Questions remain about whether IFX TDM improves outcomes.* Methods*. Patients with IBD who had IFX TDM due to concerns for loss of response were considered for inclusion. Serum IFX trough concentration and anti-drug antibody (ADA) concentrations were measured. Patients were grouped by TDM results: group 1, low IFX/high ADA; group 2, low IFX/low ADA; group 3, therapeutic IFX. Changes in management were analyzed according to groupings; remission rates were assessed at 6 months.* Results*. 71 patients were included of whom 37% underwent an appropriate change in therapy. Groups 1 (67%) and 2 (83%) had high adherence compared to only 9% in group 3. At 6 months, 57% had achieved remission. More patients who underwent an appropriate change in therapy achieved remission, though this did not reach statistical significance (69% versus 49%; *P* = 0.098).* Conclusions*. A trend towards increased remission rates was associated with appropriate changes in management following TDM results. Many patients with therapeutic IFX concentrations did not undergo an appropriate change in management, potentially reflecting a lack of available out-of-class options at the time of TDM or due to uncertainty of the meaning of the reported therapeutic range.

## 1. Introduction

Infliximab (IFX), a monoclonal antibody targeting antitumor necrosis factor alpha (TNF*α*), has become an established therapy for inducing and maintaining remission in inflammatory bowel disease (IBD) [1–3] and is routinely used in both Crohn's disease (CD) and ulcerative colitis (UC) [4]. Unfortunately, a high proportion of patients who initially respond to IFX will subsequently suffer a disease relapse [5], termed secondary nonresponse. The mechanism of loss of response is poorly understood, but mounting evidence suggests that the formation of IFX anti-drug antibodies (ADA) and pharmacokinetic variation between patients may play an important role [6–9].

**Figure 1:**
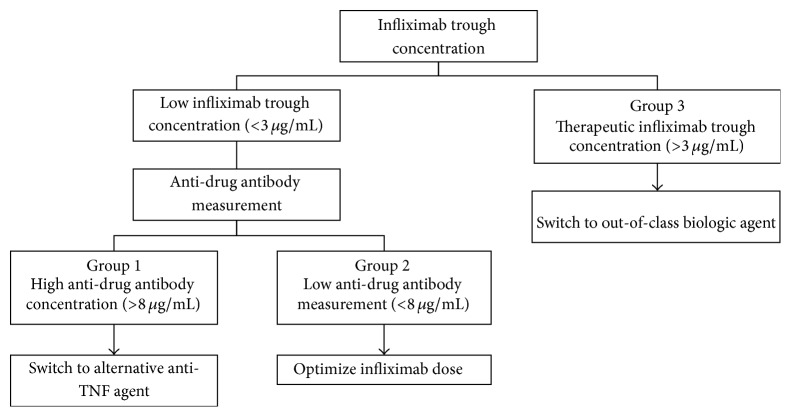
Therapeutic drug monitoring decision algorithm in inflammatory bowel disease patients on infliximab with concerns for loss of response.

Historically, physicians treating patients with secondary loss of response have relied on intuition and pragmatism to choose between dose optimization of IFX [10] or switching to an alternative biologic agent [11]. Recently, with the emergence of laboratory testing for IFX and ADA in serum, patients who suffer loss of response to IFX have guidance in making an objective therapeutic decision. It has been demonstrated that patients with low IFX trough concentrations and the presence of significant amounts of ADA generally have worse clinical outcomes, including lower rates of clinical response and poorer mucosal healing [6, 12]. Based on trough serum IFX and ADA concentrations, patients with secondary loss of response have been divided into three groups: group 1, high ADA and low IFX; group 2, low ADA and low IFX; and group 3, therapeutic IFX [13]. Based on these groupings, benefit has been demonstrated when patients in group 1 switch to an alternative anti-TNF*α* agent, group 2 undergo IFX dose optimization, and group 3 switch to an out-of-class biologic agent if available [14–16] (Figure 1).

Of note, these groupings hinge on the definitions of the IFX therapeutic window as well as the cut-off for high ADA concentrations, which will vary by the assay used for testing. For various enzyme-linked immunosorbent assays (ELISAs), recent studies have applied an IFX therapeutic range of 3 to 7 *μ*g/mL [12, 17–19] and a “high” ADA concentration of >8 *μ*g/mL [17]. Notably, in a small sample comparison study of ELISA methods, including in-house and commercial kits, highly variable results were obtained [20]. Moreover, questions remain regarding whether these thresholds can be generalized to all patients and all forms of disease, or if more personalized or disease-specific approaches should be sought. Patients in group 1 may have a loss of response related to immunogenicity of anti-TNF agents [21], and studies have shown a benefit in maintaining response to anti-TNF agents with the addition of an immunomodulator agent [2]. Patients in group 3 (therapeutic IFX levels) have been suggested to be further separated by presence of ADA using newer drug-tolerant assays as identification of the positive ADA subgroup confers higher likelihood of active disease [22–24].

Our objective was to retrospectively evaluate physician adherence to IFX therapeutic drug monitoring (TDM) guidelines among patients with concerns for secondary loss of response. Specifically, we sought to explore clinicians' decision making in response to trough IFX concentration and ADA serum concentrations and whether a correct decision in response to the TDM results was associated with improved rates of remission in patients suffering from IBD.

## 2. Methods

### 2.1. Study Participants

From 11/1/2014 to 6/30/2015, consecutive patients aged ≥19 years with histologically confirmed IBD who had been on a maintenance-dosing schedule of IFX and who had undergone IFX TDM due to concerns for secondary loss of response on an outpatient basis (Pacific Gastroenterology Associates, St. Paul's Hospital, Vancouver, Canada) were considered for study inclusion. Based on a study on IFX TDM [14], a concern for loss of response was defined as ≥ one of following criteria: (1) symptoms [CD: Harvey-Bradshaw Index > 4, UC: partial Mayo Score > 2 or individual subscore > 1]; (2) the presence of endoscopic evidence of disease; (3) biochemical evidence of disease [fecal calprotectin > 100 *µ*g/g, elevated C-reactive protein (CRP) > 5 mg/L]. Recognizing that controversy exists with respect to optimal cut-off values for fecal calprotectin, a cut-off value of 100 *μ*g/g was chosen rather than 50 *μ*g/g or 250 *μ*g/g to optimize both the sensitivity and specificity of the test (0.84 and 0.66, resp., at 100 *μ*g/g) [25, 26]. Participants were excluded if they had incomplete baseline clinical data or follow-up data. Nine physicians were involved in clinical decision making and patient assessments. All nine were gastroenterologists working at an outpatient gastroenterology practice in Vancouver, Canada. Two of the nine physicians underwent advanced IBD training.

### 2.2. Laboratory Protocol

All specimens were collected according to standard operating hospital or clinic procedures. For TDM, specimen collection occurred immediately (preferred) or less than 2 weeks prior to the next infusion.

All testing was performed by Alberta Health Services using clinically validated assays. For IFX drug concentration testing, an enzyme-linked immunosorbent assay (Immundiagnostik AG, Germany) was used. This ELISA quantifies free IFX in serum, that is, IFX not bound to ADA or other molecules. In the first incubation step, the free IFX from the patient sample is bound to the monoclonal anti-IFX antibody coated on the ELISA plate. Then the wells are washed to remove all unbound substances. In the second incubation step, a peroxidase-labeled anti-IFX antibody is added. Tetramethylbenzidine (TMB) is used as a substrate for peroxidase. Finally, an acidic stop solution is added to terminate the reaction. The change in color (from blue to yellow) is monitored spectrophotometrically, where the yellow color is directly proportional to the concentration of free IFX.

ADA to IFX were also measured by ELISA (Immundiagnostik AG, Germany). The ELISA quantifies the amount of free antibodies against IFX. In a first incubation step, the free anti-IFX antibodies from the patient's sample are bound to IFX coated on the ELISA plate, followed by a wash step. In a second incubation, peroxidase-labeled IFX is added, followed by a wash step. TMB is then added, followed by an acidic solution. The change in color (from blue to yellow) is monitored spectrophotometrically. The intensity of the color is directly proportional to the amount of free ADA present in the sample.

IFX concentrations in the range of 3 to 7 *μ*g/mL were considered in the therapeutic range. Specimens with IFX concentration <3 *μ*g/mL were reflexed for ADA testing. The limit of quantitation for ADA was 2 *μ*g/mL, with concentrations >8 *μ*g/mL being considered clinically significant.

### 2.3. Outcomes

Our primary outcome was the percentage of individuals in remission at 6 months after clinical decision making, guided by current recommendations for the interpretation of TDM among individuals with IBD and concern for secondary loss of response to IFX [13]. Remission was defined by symptoms [Harvey-Bradshaw Index of ≤ 4 (for individuals with CD) or a partial Mayo Score of ≤ 2 with no individual subscore > 1 (for individuals with UC)], no biochemical evidence of disease activity [fecal calprotectin < 100 *µ*g/g, CRP < 5 mg/L], and no endoscopic evidence of significant ulceration if endoscopy was performed at 6-month follow-up [14].

### 2.4. Statistical Analysis

Data were expressed as percentages for categorical variables and mean values for continuous variables, unless otherwise specified. SAS 9.4 (SAS Institute, Cary, North Carolina, USA) was used for statistical analysis. Chi-square tests for categorical variables and* t*-tests for continuous variables were used to assess study results. *P* values were calculated as 2-tailed and a value of <0.05 was interpreted as significant.

## 3. Results

### 3.1. Patient Characteristics

In total, 71 patients were included for analysis. Forty-four patients had CD and 27 had UC. The mean age was 38 years (19–81 years) and 52% were male. The mean duration of IBD at the time of TDM was 141 months (±86 months). Smoking data was available in 43 patients, five of whom were regular smokers (12%). The mean duration of IFX therapy, after induction, at the time of TDM was 38 months (±31 months). At the time of TDM, 45 patients (63%) had objective evidence of disease, defined by either endoscopic evidence, elevated fecal calprotectin >100 *µ*g/g, or elevated CRP >5 mg/L. Nineteen patients (27%) were on concomitant immunosuppression (IFX therapy alongside either azathioprine or methotrexate). The mean time to follow-up after TDM results was 6 months (2–12 months). When grouped by TDM results, there were 3 patients in group 1 (low IFX/high ADA), 25 patients in group 2 (low IFX/low ADA), and 43 patients in group 3 (therapeutic IFX) (Table 1).

### 3.2. Clinical Decision Making

**Figure 2:**
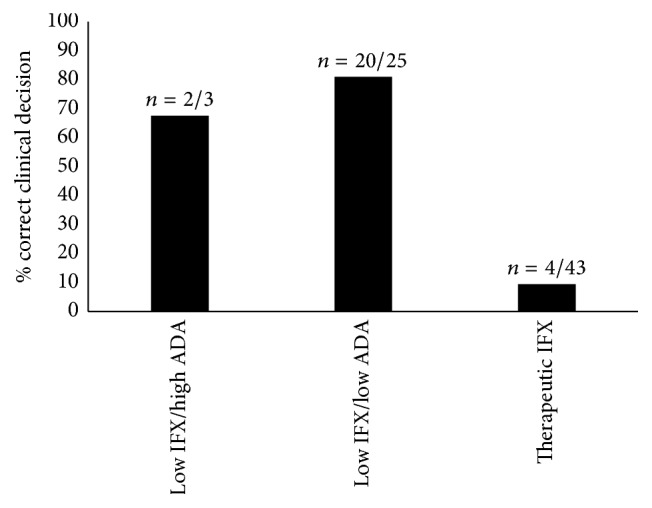
Correct clinical decision by group following infliximab therapeutic drug monitoring in inflammatory bowel disease patients with concerns for loss of response.

Twenty-six patients (37%) underwent an appropriate change in therapy after TDM results. When grouped by IBD type, 11/26 UC patients (42%) and 15/26 CD patients (34%) underwent an appropriate clinical decision after TDM. High adherence to appropriate management decisions was observed in groups 1 and 2, with 2/3 group 1 patients (67%) and 20/25 group 2 patients (80%) having correct management decisions. Conversely, only 4/43 patients (9%) in group 3 underwent an appropriate change in therapy (Figure 2). The majority of patients in group 3 underwent no change in biologic therapy (24/43 patients, 56%) or underwent dose optimization (15/43 patients, 35%). Interestingly, of the 25/43 patients in group 3 who had objective evidence of disease at the time of TDM, only 3/25 (12%) underwent an appropriate change in management to an out-of-class agent; in all 3 cases patients were switched to vedolizumab. Vedolizumab was readily available after June 1, 2015, in Canada, and only 9/71 patients (13%) in our total study population had TDM performed after this point. Of the four patients in group 3 who had an appropriate TDM decision, 3 (75%) had objective evidence of disease at TDM. Among the 39 other patients in group 3 with an inappropriate clinical decision, 22 (56%) had evidence of objective disease at the time of TDM.

### 3.3. Remission Rates

**Figure 3:**
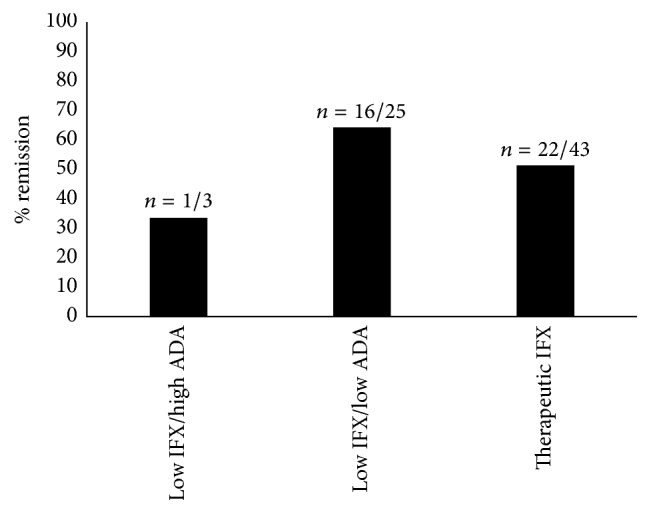
Remission at 6 months separated by group following infliximab therapeutic drug monitoring in inflammatory bowel disease patients with concerns for loss of response.

**Figure 4:**
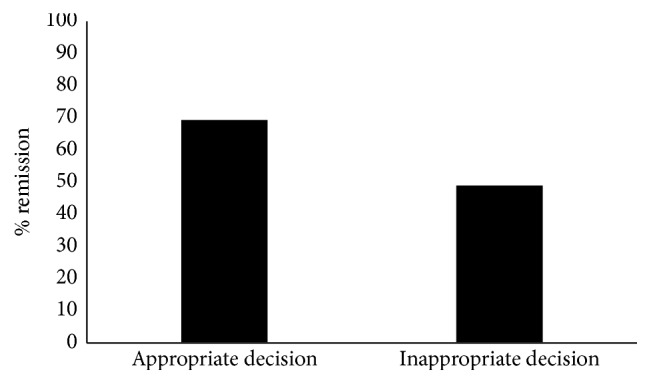
Remission at 6 months separated by clinical decision following infliximab therapeutic drug monitoring in inflammatory bowel disease patients with concerns for loss of response.

The overall remission rate at a mean follow-up time of 6 months after TDM was 57%. Fifty percent of UC and 60% of CD patients achieved remission at follow-up. Remission rates by TDM result grouping are depicted in Figure 3 (Figure 3). When comparing the impact of appropriate clinical decision making after TDM on remission rates, we observed that more patients who underwent an appropriate change in therapy achieved remission, although this did not reach statistical significance (69% versus 49%, *P* = 0.098) (Figure 4). Within group 3, 15/43 patients underwent dose optimization (35%); six of these patients achieved remission (6/15, 40%). Of the 39 patients in remission at 6 months, 22 had objective evidence of disease at the time of TDM (22/39, 56%). Among the subgroup of patients with objective evidence of disease at the time of TDM (*n* = 45), 6-month remission rates were slightly higher in those patients where a correct TDM decision was made compared to those where an incorrect TDM decision was made, but this did not reach statistical significance (56% versus 48%, *P* = 0.625).

## 4. Discussion

Although IFX is arguably the greatest development in IBD therapy over the past 2 decades, there is an annual risk for loss of response to IFX of 13% per patient year [5, 27]. This is of great significance due to the associated morbidity of IBD [28] alongside the economic ramifications of its management [28, 29], thus highlighting the importance of delineating appropriate IFX TDM use, specifically outside of a controlled setting. Therefore, the primary aim of our study was to investigate the real-world application of IFX TDM in IBD patients with concerns for secondary loss of response, specifically exploring physician adherence to TDM guidelines. Prior studies exploring IFX TDM in secondary nonresponse have demonstrated improved remission rates and mucosal healing [22, 30]. The results of this retrospective study provide insight into how clinicians in Canada are interpreting and applying TDM results in their daily practice. Our results show overall poor adherence to evidence-based TDM decision making; however, in those patients with an appropriate TDM-based decision, there was a trend towards improved rates of remission at 6 months.

To our knowledge, no published studies to date have reported on the real-world use of TDM in terms of physician adherence to appropriate TDM-based decisions and the implication this has on clinical outcomes. A recent study presenting preliminary data described a group of 22 patients with secondary nonresponse who underwent TDM. While this abstract reported remission rates and described clinician response to TDM, it does not clearly delineate between appropriate and inappropriate clinician responses to TDM and does not compare outcomes between these groups [31]. In our study, there is overall poor adherence to appropriate TDM-guided decision making. Despite this, we demonstrate a remission rate of 57% at 6-month follow-up. This may reflect the fact that not all patients included in the trial were truly secondary nonresponders to IFX. Of 39 patients in remission at 6 months, only 22 (56%) had objective evidence of disease at the time of TDM. In those patients where an appropriate TDM-based decision was made, we observed a remission rate of 69%, which trends towards a benefit compared to the observed remission rate of 49% in patients who did not undergo appropriate TDM-based decisions.

A surprising finding of the present study is the low overall rate of adherence to correct clinical decision making following TDM. This was largely driven by patients in group 3 which comprised 60% of the total study population and had an adherence rate of 9% to correct TDM decisions. The poor adherence rate in this group may be explained by several factors. A notable factor is the lack of available out-of-class biologic options in Canada during the study period, as only 13% of study patients would have had the option of switching to an approved out-of-class biologic. Four patients in group 3 who were appropriately switched to an out-of-class agent were switched to vedolizumab, and three of these patients achieved remission. Poor adherence in group 3 may also be explained by a belief among physicians that the upper limit of the current IFX therapeutic window (7 *μ*g/mL) should be increased. Other published studies have proposed a wider (3–10 *μ*g/mL) IFX therapeutic range or different cut-off values for CD and UC, leading some gastroenterologists to intensify the IFX dose in patients in group 3 [22, 32]. In a similar study by Paul et al. [30], patients with a therapeutic IFX concentration (analogous to group 3 in the present study) comprised 37% of the study population. Interestingly, each patient in group 3 of the Paul et al. study was dose-optimized and only 21% achieved remission. This is in contrast to the 52% remission rate observed in group 3 in the present study, where more than half of such patients had no change in management. In the present study, patients in group 3 who were dose-optimized achieved a 40% remission rate. Finally, patients in group 3 who had an appropriate clinical decision based on TDM had a higher rate of objective evidence of disease at the time of TDM than patients who did not have an appropriate TDM decision. It is possible that physicians treating this group sought additional evidence of disease at follow-up appointments before switching these patients to an alternative out-of-class agent. How to best manage patients in group 3 remains unclear—whether to dose-intensify or immediately switch to an out-of-class agent—and is an important area of future research.

The present study observed low immunogenicity to IFX. Three of 28 patients (11%) tested were positive for ADA. This is less than other studies, where 16% [17] and 17% [19] of patients tested were positive for ADA. The lower rate of observed immunogenicity in the present study is particularly surprising given this study's population of secondary nonresponders. The observed low immunogenicity is in the context of only 27% of study patients on concomitant immunosuppression. In the three patients who developed ADAs, none used concomitant immunosuppression. Another potential explanation may be that all patients in this study were on scheduled treatment of infliximab, which is known to be associated with less immunogenicity compared to patients where it is used episodically [33].

A limitation of the present study is the small study population, which limits the ability to generalize and interpret results. Another limitation is the difficulty of identifying secondary nonresponders. This study included patients where a concern for loss of response was raised based on clinical, biochemical, or endoscopic parameters. This may have led to the contamination of the study population with patients who did not truly lose response and had symptoms or biochemical changes for other reasons. Of note, one patient in the study exhibited loss of response based on endoscopic evidence of moderate inflammation with a Harvey-Bradshaw Index of 2 (remission). This patient had Crohn's disease and was found to have a therapeutic IFX level at TDM. This patient was appropriately switched to an out-of-class agent but did not have follow-up endoscopy and was classified as “in remission” given Harvey-Bradshaw Index of 0 and normal CRP. Exclusion of patients who did not have adequate follow-up data after TDM is another study limitation. Additionally, an average follow-up time of 6 months may not be sufficient to complete the clinical work-up when concern for loss of response is raised and thus may not reflect long-term decision-making practices. Lastly, a drug-tolerant assay was not available during the time of this study and thus group 3 could not be better characterized by the presence of ADA and analyzed within this context; dividing group 3 by presence of ADA offers a prognostic advantage and has been employed in other research protocols [34–36].

Nevertheless, the present study does capture real-world data on the impact of TDM on the care of patients with IBD and provides valuable information on the need to educate physicians on the role of appropriate decision making in response to TDM in order to optimize outcomes of infliximab treatment of IBD. Based on results from this study, physician adherence to TDM may specifically be improved by physician education around the appropriate response to patients with therapeutic IFX serum concentration who have lost response.

## 5. Conclusions

This is the first study describing the current application of IFX TDM in the Canadian landscape with a focus on adherence to TDM and impact on disease remission. Despite the low adherence to recommended clinical decisions after IFX TDM results, there was a positive yet not statistically significant trend towards improved remission rates in those with appropriate TDM-based decisions. Patients in group 3 were least likely to have their therapy appropriately changed to an out-of-class agent. This may reflect multiple factors including the lack of available alternate therapeutic options during the study period, physician beliefs regarding the upper therapeutic limit of IFX, and physician education regarding interpretation of TDM results. With the emergence of vedolizumab and other novel IBD therapeutics, there is opportunity to better understand how to treat patients with suspected loss of response who have therapeutic IFX concentrations. Further education on appropriate use of this valuable laboratory information will ensure ideal management to control disease and maximize remission rates, thereby improving patient care while maximizing resource utilization.

## Figures and Tables

**Table 1:** Characteristics of inflammatory bowel disease patients on infliximab with concerns for loss of response at time of therapeutic drug monitoring.

	Low IFX/high ADA (*n* = 3)	Low IFX/low ADA (*n* = 25)	Therapeutic IFX (*n* = 43)
Gender, male	2 (66.7%)	16 (64.0%)	19 (44.2%)
Age, years (range)	42 (31–63)	37 (19–81)	38 (19–68)
UC	1 (33.3%)	12 (48.0%)	14 (32.6%)
CD	2 (66.7%)	13 (52.0%)	29 (67.4%)
Disease duration, months ± SD	112 ± 86	110 ± 98	160 ± 101
Baseline partial Mayo Score ± SD	3.0 ± 0	4.7 ± 1.4	4.4 ± 2.0
Baseline Harvey-Bradshaw Score ± SD	3.5 ± 2.1	5.9 ± 4.3	6.7 ± 4.3
Objective evidence of disease	3 (100%)	17 (68.0%)	25 (58.1%)
Infliximab duration, months ± SD	25 ± 24	27 ± 28	45 ± 32
Concomitant immunosuppression	0 (0%)	10 (40.0%)	9 (20.9%)
